# RETRACTION: Exogenous High‐Mobility Group Box 1 Protein Injection Improves Cardiac Function after Myocardial Infarction: Involvement of Wnt Signaling Activation

**DOI:** 10.1155/bmri/9874862

**Published:** 2026-04-17

**Authors:** BioMed Research International

RETRACTION: X. Zhou, X. Hu, J. Xie, C. Xu, W. Xu, and H. Jiang, “Exogenous High‐Mobility Group Box 1 Protein Injection Improves Cardiac Function After Myocardial Infarction: Involvement of Wnt Signaling Activation,” *BioMed Research International* 2012, no. 1 (2012): 743879, https://doi.org/10.1155/2012/743879.

The above article, published online on 21 May 2012 in Wiley Online Library (wileyonlinelibrary.com), has been retracted by John Wiley & Sons Ltd. The retraction has been agreed due to figure concerns, initially identified on PubPeer [1]. Specifically, in Figure 3, subfigure Figures MI and MI‐HMGB1 (7 days) appear to contain overlapping sections, despite presenting different experimental conditions. The overlapping sections also appear to each have a different magnification, although the image key indicates that they are at the same magnification. The authors did not respond to requests for original images and an explanation.

The results and conclusions are therefore considered unreliable.

The authors did not respond when notified of the retraction.
